# A 19-Month-Old Girl With Acute Choledocholithiasis: A Case Report

**DOI:** 10.7759/cureus.83433

**Published:** 2025-05-03

**Authors:** Luis C Gonzalez Isoba, Maya Barrant, Nadiya Zafar, Christine Salib, Carolina Fiamengo

**Affiliations:** 1 Internal Medicine, Nova Southeastern University Dr. Kiran C. Patel College of Osteopathic Medicine, Fort Lauderdale, USA; 2 Medicine, Nova Southeastern University Dr. Kiran C. Patel College of Osteopathic Medicine, Fort Lauderdale, USA; 3 Pediatrics, Broward Health Medical Center, Fort Lauderdale, USA

**Keywords:** abdominal pain, cholecystitis, choledocholithiasis, cholelithiasis, gallstone pancreatitis, omphalocele

## Abstract

Cholelithiasis, or gallstone(s), is a leading cause of healthcare utilization in the United States. It is more common in adults but can occur in the pediatric population as well. The following is a case report of choledocholithiasis in a 19-month-old girl.

A 19-month-old girl with abdominal pain presented to the emergency department (ED) after being found to have cholelithiasis on an outpatient abdominal ultrasound (US). Three days prior to presentation, the patient was seen by her pediatrician for fussiness, decreased oral intake, and non-bloody, non-bilious emesis. She was diagnosed with a suspected urinary tract infection (UTI) and prescribed amoxicillin-clavulanate for empiric treatment. The following day, the patient returned to her pediatrician for worsening abdominal pain; she was given one dose of intramuscular ceftriaxone and scheduled for outpatient abdominal US.

Her past medical history is significant for omphalocele status post-surgical correction, several congenital cardiac defects, bilateral small kidneys, and poor weight gain. The patient has a normal chromosomal microarray and no family history of hepatobiliary/pancreatic disease.

In the ED, the patient was afebrile and hemodynamically stable. Physical examination was significant for mild hepatomegaly, mild abdominal tenderness without peritoneal signs, and the presence of a well-healed surgical scar on the abdomen with an underlying abdominal hernia. Laboratory tests were significant for leukocytosis of 14.5×10^3^/microliter (mcL), elevated gamma-glutamyl transferase (GGT) of 305 unit/L (U/L), aspartate aminotransferase (AST) of 86 U/L, alanine aminotransferase (ALT) of 343 U/L, total bilirubin of 2.3 milligram/deciliter (mg/dL), direct bilirubin of 1.6 mg/dL, and lipase of 1,726 U/L. Abdominal US revealed several gallstones and mild to moderate intra- and extrahepatic biliary ductal dilatation likely due to a stone in the distal common bile duct (CBD). Pediatric surgery and gastroenterology recommended admission for pain management and magnetic resonance cholangiopancreatography (MRCP).

After admission, the patient was started on ursodiol and piperacillin/tazobactam. MRCP showed a common hepatic duct measuring 13 mm and a 9×5 mm stone in the distal common bile duct. Due to a lack of available outside facilities with the capability to perform endoscopic retrograde cholangiopancreatography (ERCP) in a pediatric patient, medical management was pursued. Throughout her admission, the patient improved clinically, laboratory studies became normal, and pain was controlled. Repeat US showed persistent biliary dilation with cholelithiasis. The patient was cleared for discharge on ursodiol and amoxicillin-clavulanate and close follow-up with pediatrician, pediatric surgeon, and pediatric gastroenterologist.

Follow-up US performed two weeks after discharge showed interval resolution of intra- and extrahepatic biliary duct dilatation and cholelithiasis without evidence of cholecystitis.

Abdominal pain accounts for 5%-10% of all pediatric ED visits, and although cholelithiasis and choledocholithiasis are rare in the pediatric population, as this case demonstrates, it is an important differential diagnosis. Observation is the recommended management for asymptomatic patients as most cases spontaneously resolve. Patients with clinical signs or laboratory abnormalities can be treated medically, with ERCP, or with cholecystectomy.

## Introduction

Cholelithiasis, or gallstone(s), is a leading cause of healthcare utilization in the United States [1], with incidence tripling over the last three decades [2]. While 6%-9% of adults develop gallstones [3], prevalence in children ranges from 0.13% to 0.22% [4]. In the pediatric population, risk factors for cholelithiasis include obesity, family history, hemolytic disease, hepatobiliary disease, prolonged parenteral nutrition, ileal resection, and ceftriaxone use [4,5]. The following is a case report of choledocholithiasis in a 19-month-old girl.

## Case presentation

A 19-month-old female patient with abdominal pain presented to the emergency department (ED) after being found to have cholelithiasis on an outpatient abdominal ultrasound (US). Three days prior to presentation, the patient was seen by her pediatrician for fussiness, decreased oral intake, and non-bloody, non-bilious emesis. She was diagnosed with a suspected urinary tract infection (UTI) and prescribed amoxicillin-clavulanate for empiric treatment. The following day, the patient returned to her pediatrician for worsening abdominal pain; she was given one dose of intramuscular ceftriaxone and scheduled for an outpatient abdominal ultrasound.

In terms of past medical history, the patient was born full term via scheduled cesarean section due to omphalocele on fetal ultrasound. After delivery, she required positive pressure ventilation (PPV) and was placed on nasal continuous positive airway pressure (CPAP) prior to admission to the neonatal intensive care unit (NICU). During her NICU stay, the patient was found to have a murmur auscultated on examination. Echocardiogram revealed multiple congenital cardiac defects, including a small muscular ventricular septal defect (VSD), a large bidirectional patent ductus arteriosus (PDA), a patent foramen ovale (PFO), a dysplastic aortic valve, and persistent left superior vena cava (PLSVC), as well as transitional infra-systemic pulmonary hypertension. Laboratory studies at that time showed high levels of aldosterone and renin. Renal ultrasound was completed, showing bilateral small kidneys. She initially had poor weight gain, which improved after increasing caloric intake. Omphalocele was repaired prior to discharge without complications. The patient has a normal chromosomal microarray and no family history of hepatobiliary or pancreatic disease.

**Figure 1:**
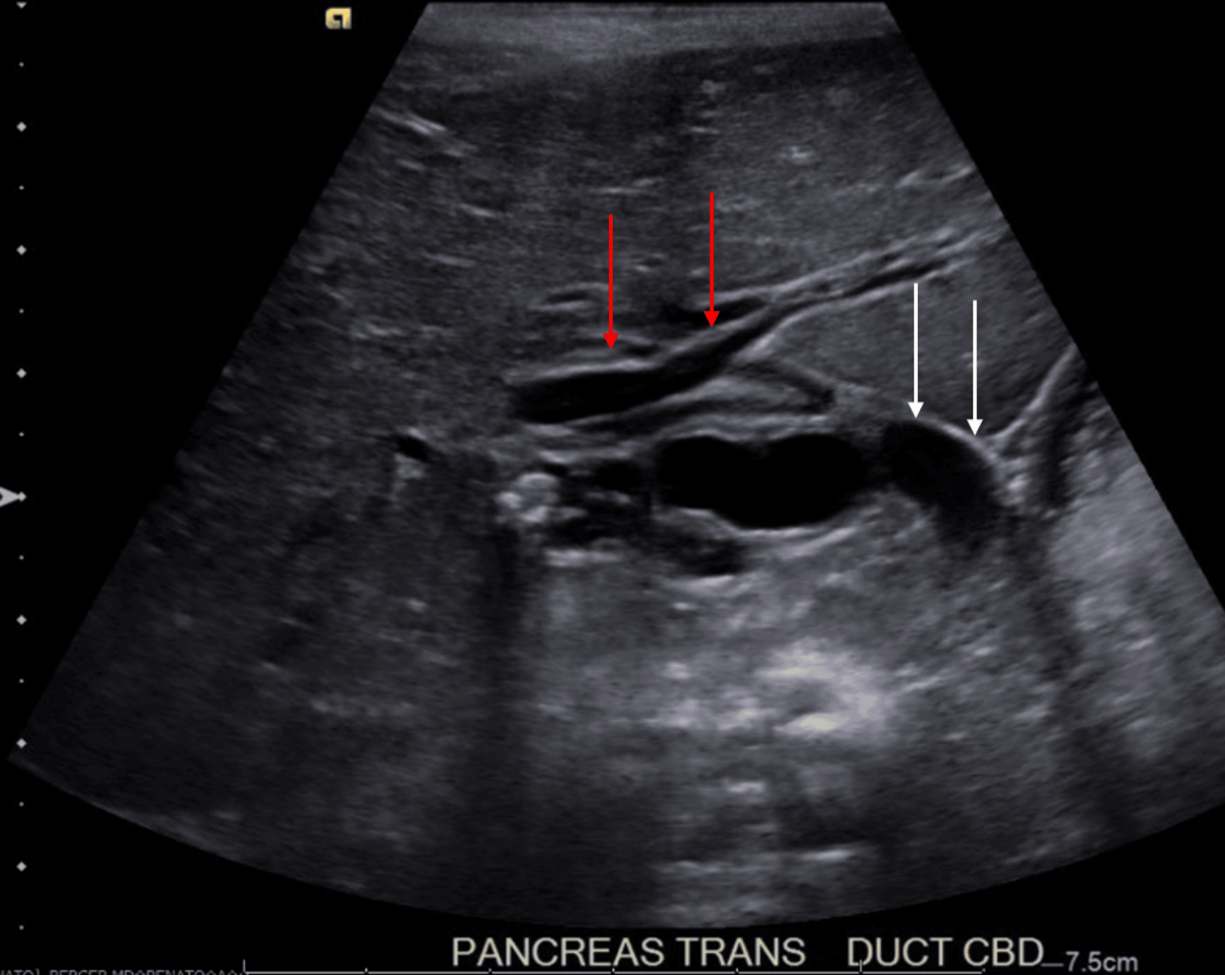
Initial abdominal ultrasound Abdominal ultrasound (pancreas transverse view) showing several stones within the gallbladder without gallbladder wall thickening, mild to moderate intra- and extrahepatic biliary ductal dilatation with the common bile duct measuring 9 mm and an ovoid lesion measuring 1.5×0.6 cm in the distal common bile duct. Red arrows point to intrahepatic biliary ductal dilatation. White arrows point to dilated gallbladder and extrahepatic biliary ductal dilatation.

In the ED, the patient was afebrile and hemodynamically stable. Her physical examination findings included mild hepatomegaly, mild abdominal tenderness without peritoneal signs, and the presence of a well-healed surgical scar on the abdomen with an underlying abdominal hernia. Laboratory studies were significant for leukocytosis, transaminitis, direct hyperbilirubinemia, and hyperlipasemia. Specific values are displayed in Table 1. Abdominal US showed several gallstones and mild to moderate intra- and extrahepatic biliary ductal dilatation likely due to a stone in the distal common bile duct (CBD) (Figure 1).

**Table 1:** Initial laboratory values upon presentation WBC: white blood cells, GGT: gamma-glutamyl transferase, AST: aspartate aminotransferase, ALT: alanine aminotransferase, T. Bili: bilirubin (total), D. Bili: bilirubin (direct), mcL: microliter, U/L: unit/liter, mg/dL: milligram/deciliter

Laboratory value	Level	Reference values
WBC	14,500 mcL	4,000-11,000 mcL
GGT	305 U/L	<50 U/L
AST	86 U/L	10-40 U/L
ALT	343 U/L	7-56 U/L
T. Bili	2.3 mg/dL	0.1-1.2 mg/dL
D. Bili	1.6 mg/dL	0.0-0.3 mg/dL
Lipase	1,726 U/L	10-140 U/L

Pediatric surgery and gastroenterology were consulted and recommended admission for pain management and magnetic resonance cholangiopancreatography (MRCP) to detail the biliary and pancreatic systems.

**Figure 2:**
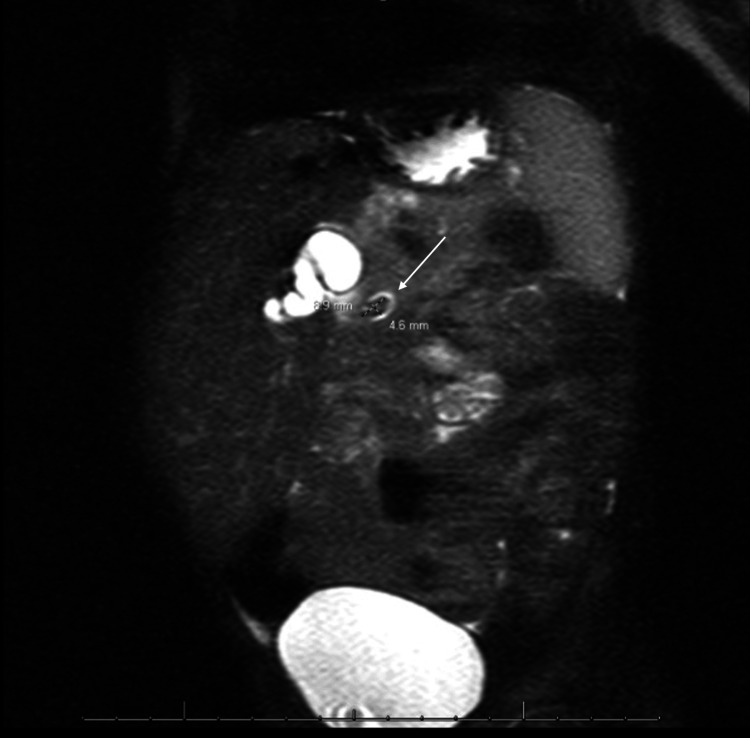
MRCP MRCP showing the gallbladder distended with multiple gallstones, trace amount of free fluid adjacent to the liver, and moderate intra- and extrahepatic biliary ductal dilatation with the common hepatic duct measuring 13 mm in diameter and an ovoid filling defect measuring 9×5 mm in the distal common bile duct (white arrow). MRCP: magnetic resonance cholangiopancreatography

After admission, the patient was started on ursodiol, piperacillin-tazobactam, and maintenance intravenous fluids containing dextrose. A nothing by mouth (NPO) order was also placed in preparation for imaging under sedation. The following morning, MRCP was completed, showing the common hepatic duct measuring 13 mm and a 9×5 mm stone in the distal common bile duct (Figure 2).

**Figure 3:**
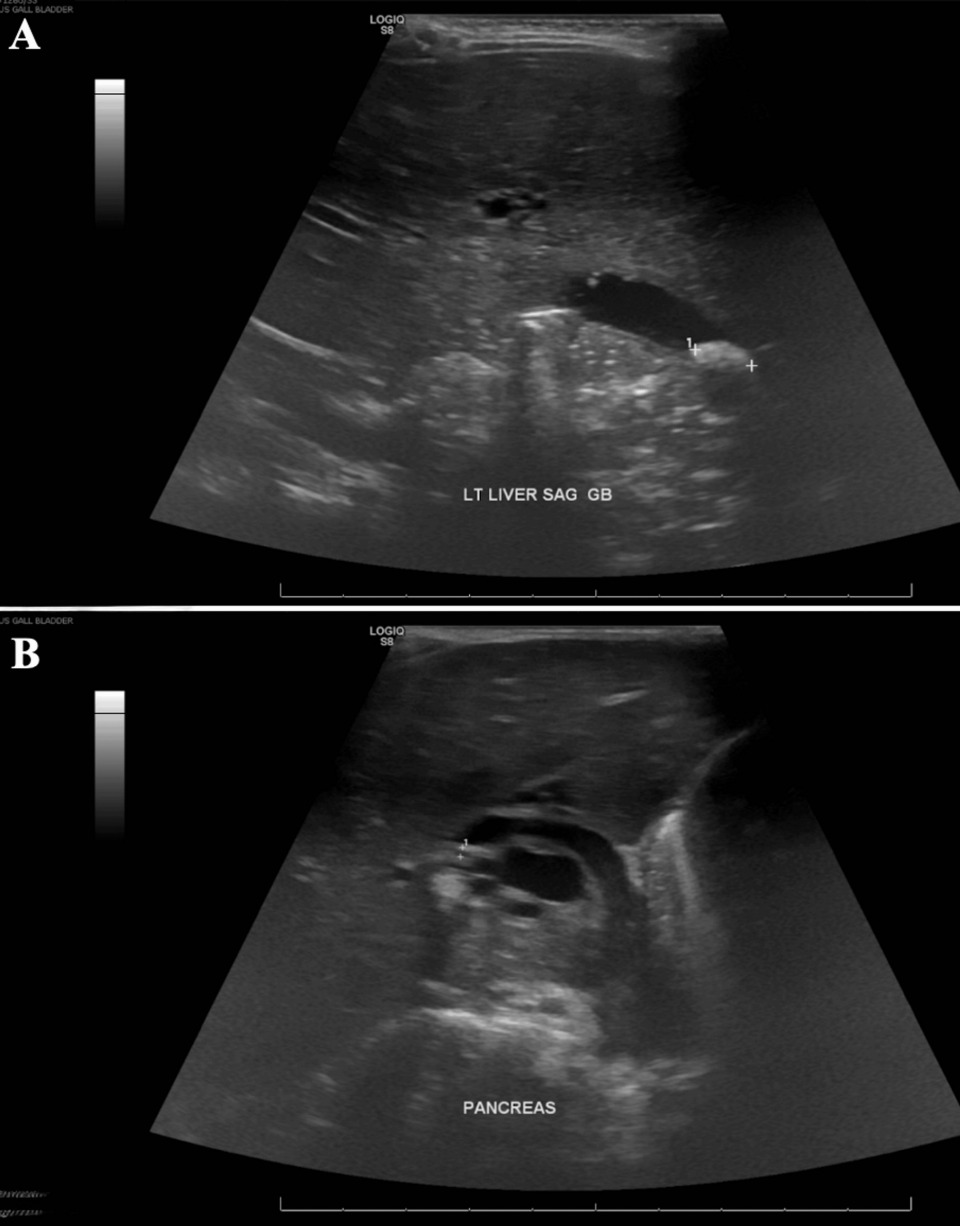
Repeat abdominal ultrasound Repeat abdominal ultrasound (A: liver sagittal view, B: pancreas view) showing the intrahepatic ducts and CBD remaining dilated with multiple gallstones, the largest of which measuring 9 mm. No gallbladder wall thickening. CBD: common bile duct

Due to a lack of pediatric interventional gastroenterologists and appropriate equipment at our institution, efforts to transfer the patient to an outside facility with the capability to perform endoscopic retrograde cholangiopancreatography (ERCP) in pediatric patients were started. Since admission, the patient had significant clinical improvement, laboratory abnormalities resolved, and pain was well controlled. Repeat ultrasound (Figure 3) had persistent biliary dilation with cholelithiasis. Due to a lack of availability of outside facilities with ERCP and patient clinical improvement, a decision was made to continue medical management. The patient was cleared for discharge on ursodiol and amoxicillin-clavulanate and close follow-up with pediatrician, pediatric surgeon, and pediatric gastroenterologist.

**Figure 4:**
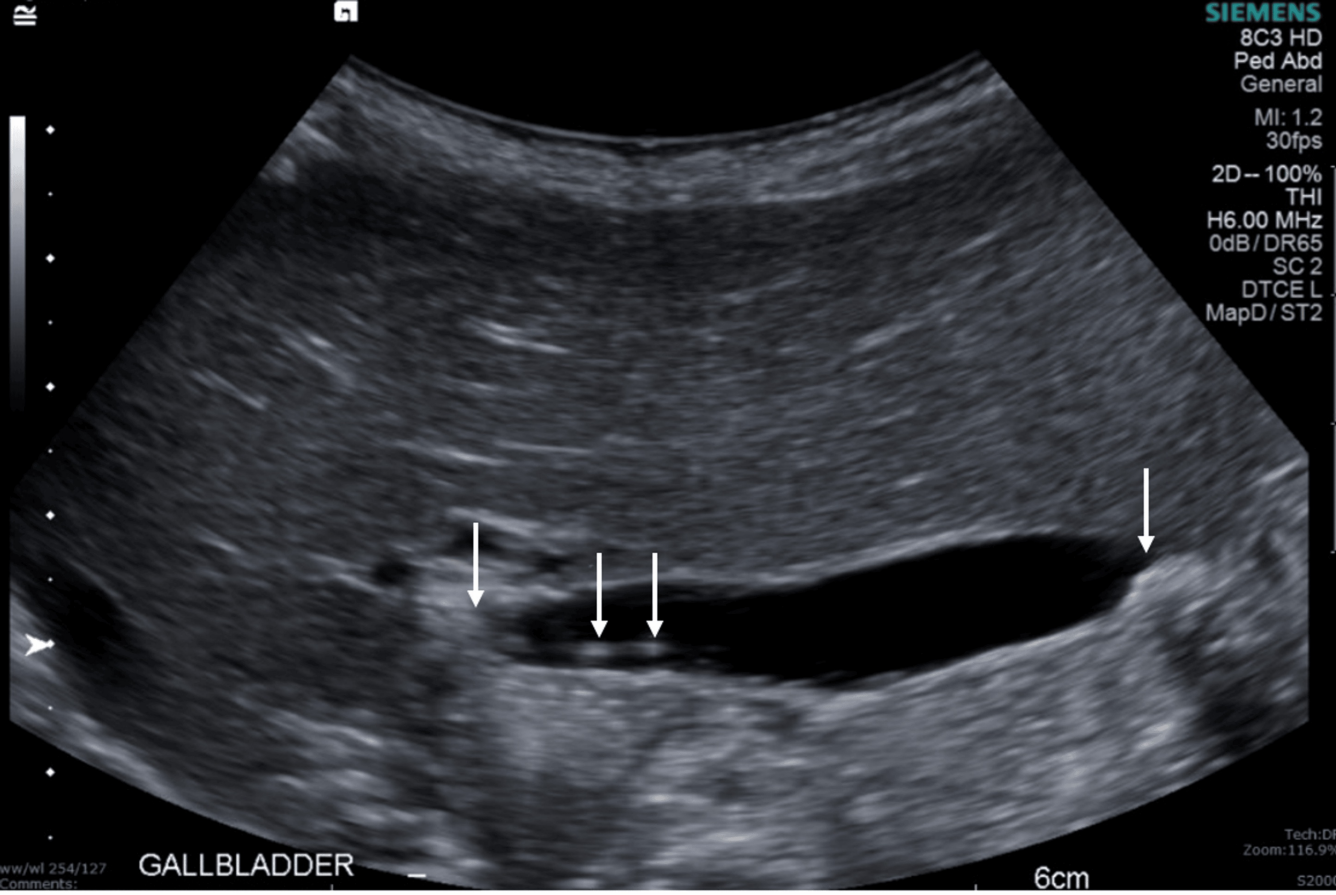
Follow-up abdominal ultrasound Follow-up abdominal ultrasound (gallbladder view) showing interval resolution of previously seen intra- and extrahepatic biliary duct dilatation and cholelithiasis without cholecystitis, including a 6 mm gallstone in the neck of the gallbladder.

Follow-up US performed two weeks after discharge (Figure 4) showed interval resolution of intra- and extrahepatic biliary duct dilatation and cholelithiasis without evidence of cholecystitis.

## Discussion

Cholelithiasis, or gallstone(s), is a prevalent gastrointestinal pathology in the United States, presenting in either symptomatic or asymptomatic forms. The term "cholelithiasis" broadly refers to the presence of gallstones, which can be localized to various regions of the gastrointestinal tract, including the gallbladder (cholecystolithiasis) and the common bile duct (choledocholithiasis). The development of gallstones is influenced by multiple factors, including genetic predisposition, hormonal influences, metabolic disorders, and lifestyle factors [1].

Epidemiology

Gallstone disease has a multifactorial etiology with several established risk factors, including genetic predisposition, obesity, and rapid weight loss. Familial aggregation studies suggest a significant hereditary component (30%-40%) to gallstone susceptibility. The occurrence of fetal cholelithiasis further supports a potential genetic basis for gallstone formation [6]. Moreover, the influence of sex hormones on hepatic bile secretion and gallbladder function may account for the observed increased risk of cholelithiasis among females, compounded by the use of oral contraceptives and estrogen replacement therapy [7]. Additional risk factors include metabolic conditions such as dyslipidemia, diabetes mellitus, metabolic syndrome, and chronic diseases such as liver disease, Crohn's disease, cystic fibrosis, and sickle cell disease. Dietary factors, particularly high intake of cholesterol, fatty acids, and carbohydrates, also contribute to gallstone pathogenesis. Furthermore, medications such as octreotide, ceftriaxone, thiazide diuretics, and statins are associated with an elevated risk of gallstone formation.

The prevalence and typology of gallstones vary by ethnicity and geographic region. In North America, cholesterol gallstones predominate, particularly among Indigenous Americans (64.1% of women and 29.5% of men), who exhibit the highest prevalence rates, followed by White (16.6% of women and 8.6% of men) and Black populations (13.9% of women and 5.3% of men). Prevalence also increases with age, especially after age 40 [7]. An increase in gallstone prevalence has been noted in pediatric populations, mirroring trends observed in adults. Pediatric cases often correlate with obesity and genetic factors, such as cholecystokinin (CCK-1) receptor deletions and mutations in the cholesterol 7-alpha hydroxylase gene, which are linked to increased risk for stone formation. Poor gallbladder contractility, impaired function or release of CCK, and excessive release of fibroblast growth factor 19 are also linked to increased stone formation [8]. Most individuals with gallstones, whether adult or pediatric, are asymptomatic, with estimations of around 40%-51% of children with gallstones being asymptomatic [7].

Pathophysiology

The composition of gallstones indicates the reason for their formation, the two main types being cholesterol and pigment stones. Cholesterol gallstones are mainly composed of cholesterol, ranging from 70% to 100%, and other components such as bilirubin, protein, and calcium carbonate [1]. They are due to excessive cholesterol production by liver cells and reduced gallbladder motility or impaired emptying [4]. Pigmented stones are formed from calcium bilirubinate, which is a calcium salt from unconjugated bilirubin [1]. In conditions with elevated heme turnover, bilirubin levels in bile may exceed normal concentrations. Bilirubin can subsequently precipitate and crystallize, leading to pigmented stone formation [4].

When stones obstruct the cystic duct or the bile duct, the classic symptoms of cholelithiasis occur. If a stone temporarily obstructs the opening of the cystic duct, transient biliary pain occurs, known as cholelithiasis. If the obstruction becomes more frequent and a large stone lodges in the neck of the gallbladder, acute cholecystitis may occur. In some cases, a gallstone may pass through the cystic duct but then become stuck in the common bile duct (CBD), and the condition is termed choledocholithiasis [4].

Acute gallstone pancreatitis is due to fluid backup and increased pressure within the pancreatic ducts, along with the activation of pancreatic enzymes in situ. It is due to a gallstone that managed to get through the cystic duct and CBD but is not able to traverse the ampulla of Vater. On occasion, larger gallstones may perforate the gallbladder wall, which forms a fistula between the gallbladder and either the small or large bowel. This may result in bowel obstruction or ileus [4].

Presentation

Most patients with gallstones are asymptomatic, with cholelithiasis being an incidental finding on imaging. Symptomatic patients typically present with biliary colic, an intense, constant pain in the right upper quadrant. Pain is often triggered by eating fatty meals. There is also associated nausea and vomiting. Atypical symptoms include belching, early satiety, regurgitation, abdominal distention/bloating, epigastric or retrosternal burning, nausea or vomiting alone, chest pain, and nonspecific abdominal pain [9]. In the case of our patient, because she was only 19 months old and unable to communicate her symptoms, her presentation had a broad differential. She presented with abdominal pain, fussiness, several episodes of emesis, and decreased oral intake for 4-5 days. Her parents stated that this was the first time these symptoms had occurred, and they did not recall any specific meal that might have triggered them.

Diagnostic evaluation

The general approach to diagnosing gallstone disease in adults includes a good history, physical examination, laboratory tests, and imaging. Uncomplicated cholelithiasis should be suspected in a patient with biliary colic, unremarkable physical examination, and unremarkable laboratory results; abnormal blood tests may indicate complications of cholelithiasis, such as choledocholelithiasis [9]. Choledocholelithiasis presents similarly to symptomatic cholelithiasis, but elevated liver enzymes in a primarily cholestatic pattern are seen on laboratory results. Compared with aspartate aminotransferase (AST) and alanine aminotransferase (ALT), there is disproportionate elevation of alkaline phosphatase, gamma-glutamyl transferase, and bilirubin [10].

The first-line imaging for biliary colic is transabdominal ultrasonography to detect stones or sludge [8]. Stones in the gallbladder have an echogenic acoustic shadow, but stones in the CBD may be harder to detect due to overlying bowel gas [8]. If a patient presents with biliary colic but no gallstones were found on ultrasonography, then imaging is repeated in a few weeks to detect missed gallstones. If repeat ultrasonography is also negative, an endoscopic ultrasound can be performed. This may be helpful for the diagnosis of choledocholelithiasis [8]. If this is also negative, bile microscopy can be used to detect sludge or microlithiasis. ERCP can be used for both diagnostic and therapeutic purposes. ERCP has a success rate of 95% for the treatment of CBD stones in pediatric cases. It is the favored method for diagnosing congenital abnormalities in the pancreaticobiliary ducts [8]. However, evaluation with additional imaging may not be necessary, and the decision to use these alternative methods must consider patient preference, availability of expertise, and risk of adverse outcomes [9].

Management

Asymptomatic stones do not require treatment. However, if a patient has symptomatic gallstones, there are several methods to manage them. There are three main non-surgical methods for the management of gallstones in adults: ursodeoxycholic acid (UDCA), extracorporeal shock wave lithotripsy (ESWL), and cholecystolithotomy [10]. UDCA is a secondary bile acid found at low concentration in human bile. UDCA therapy is only effective on cholesterol stones, and it is the most commonly used agent. However, there are limited studies showing the efficacy of this treatment in pediatric populations [8]. ESWL was introduced in the 1980s as an alternative method, but initial studies showed 60% recurrence rates at 10 years. A more recent study saw recurrence rates of 43% at five years. There has been only a single report of ESWL being successful in the treatment of gallstones in a 12-year-old girl, who was stone-free and asymptomatic 18 months post-intervention [8]. The final non-surgical method, cholecystolithotomy, involves simple removal of gallstones while leaving the gallbladder intact. It was another alternative introduced in the 1980s that saw a recurrence rate of >40% at 10 years. The largest study of cholecystolithotomy use in pediatric populations saw 10 children receive the treatment, with 30% having a recurrence. Nonsurgical treatment for gallstone disease in both adults and children is associated with high recurrence rates, with many patients ultimately choosing to have cholecystectomy. Current literature does not support the use of non-surgical methods for the treatment of gallstones in children [8].

Cholecystectomy is the most common treatment for cholelithiasis in both adults and children. Both open and laparoscopic are acceptable approaches to be used, although the laparoscopic technique is the most commonly used and preferred method [11]. Retrospective studies have shown that laparoscopic cholecystectomies are associated with shorter hospital stays, reduced analgesia requirements, and lower treatment costs compared to open cholecystectomies, thus making it the standard treatment for symptomatic gallstones in children [6,8].

Similarly to cholelithiasis, surgical management is indicated for patients with symptomatic choledocholelithiasis. Based on a 20-year retrospective study by Pogorelić et al., all pediatric patients with symptomatic biliary colic should undergo abdominal ultrasonography. If there is no clinical evidence of choledocholelithiasis or dilation of the CBD, then patients should be scheduled for elective laparoscopic cholecystectomy. If there is dilation of the CBD and clinical signs of choledocholelithiasis, then MRCP should be performed. If stones are detected, the next step would be laparoscopic common bile duct exploration (LCBDE) in which a laparoscopic cholecystectomy and exploration of the CBD are done. Once exposed, the stone should be removed with the Dormia basket or crushed with a laser and removed with the Dormia basket, if the former technique is not possible. ERCP should be done in the case that none of these techniques are successful [12]. Although ERCP and LCBDE are accepted techniques for the management of choledocholelithiasis in pediatric populations, LCBDE provides definitive treatment in a single procedure under anesthetic, while ERCP is done preoperatively or postoperatively with laparoscopic cholecystectomy. However, there has been a trend in the last decade toward favoring ERCP usage over LCBDE [13]. Laparoscopic techniques are the preferred method of treatment [8].

Our patient

This patient's presentation is interesting as cholelithiasis and choledocholelithiasis do not occur often in children, and there is a lack of literature for these conditions at such a young age. The prevalence in children ranges from 0.13% to 0.22% [3]. The most common risk factors for cholelithiasis were not present in this patient. Her history was negative for obesity, hemolytic disease, hepatobiliary disease, prolonged parenteral nutrition, and a family history of cholelithiasis. Her parents reported that her diet consisted of the same foods they ate, which included rice, beans, and different kinds of meat. High-fat foods that might have been present in the patient's diet could have been the cause of her gallstone development. In addition, the patient received a dose of ceftriaxone for a presumed UTI before presentation, and there have been reports of an association between ceftriaxone administration in children and cholelithiasis [5]. Overall, given her history, her case seems to be of idiopathic etiology.

## Conclusions

Abdominal pain accounts for 5%-10% of all pediatric emergency department visits, and although cholelithiasis and choledocholithiasis are rare in the pediatric population, as this case demonstrates, it is an important differential diagnosis. Observation is the recommended management for asymptomatic patients, as most cases spontaneously resolve; patients with clinical signs or laboratory abnormalities can be treated medically, with ERCP, or with cholecystectomy.
